# Supplementary material for: Production of Plant-Derived Japanese Encephalitis Virus Multi-Epitope Peptide in Nicotiana benthamiana and Immunological Response in Mice
Source: Int J Mol Sci. 2023 Jul 19;24(14):11643. doi: 10.3390/ijms241411643 (PMC10380836; doi:10.3390/ijms241411643)
Supplement: Supplementary file 1 [file ijms-24-11643-s001.zip › JEV-MEP_Supplementary Figures.pptx]

## Slide 1
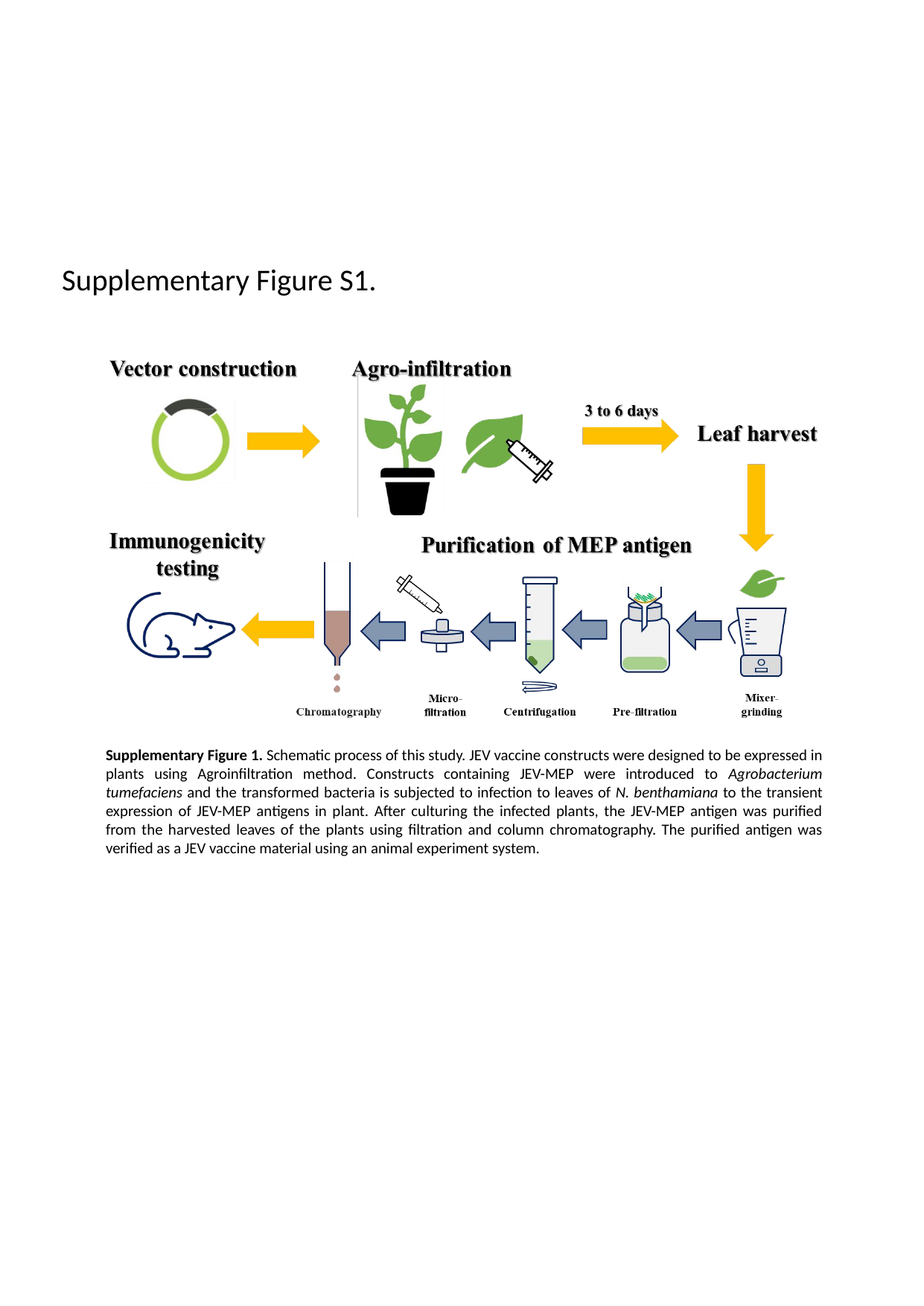

Supplementary Figure S1.
Supplementary Figure 1. Schematic process of this study. JEV vaccine constructs were designed to be expressed in plants using Agroinfiltration method. Constructs containing JEV-MEP were introduced to Agrobacterium tumefaciens and the transformed bacteria is subjected to infection to leaves of N. benthamiana to the transient expression of JEV-MEP antigens in plant. After culturing the infected plants, the JEV-MEP antigen was purified from the harvested leaves of the plants using filtration and column chromatography. The purified antigen was verified as a JEV vaccine material using an animal experiment system.

## Slide 2
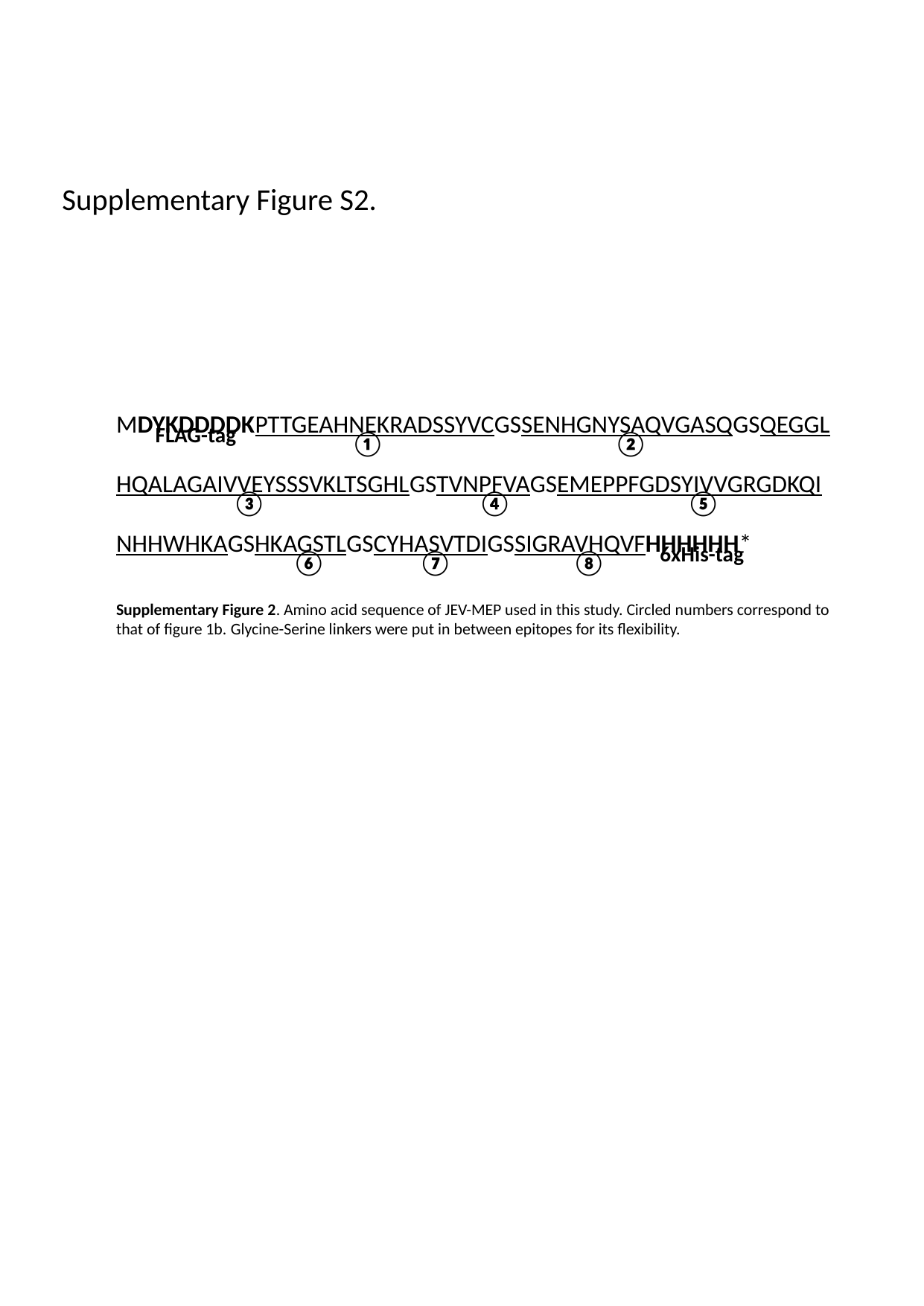

Supplementary Figure S2.
MDYKDDDDKPTTGEAHNEKRADSSYVCGSSENHGNYSAQVGASQGSQEGGLHQALAGAIVVEYSSSVKLTSGHLGSTVNPFVAGSEMEPPFGDSYIVVGRGDKQINHHWHKAGSHKAGSTLGSCYHASVTDIGSSIGRAVHQVFHHHHHH*
FLAG-tag
①
②
③
④
⑤
6xHis-tag
⑥
⑦
⑧
Supplementary Figure 2. Amino acid sequence of JEV-MEP used in this study. Circled numbers correspond to that of figure 1b. Glycine-Serine linkers were put in between epitopes for its flexibility.

## Slide 3
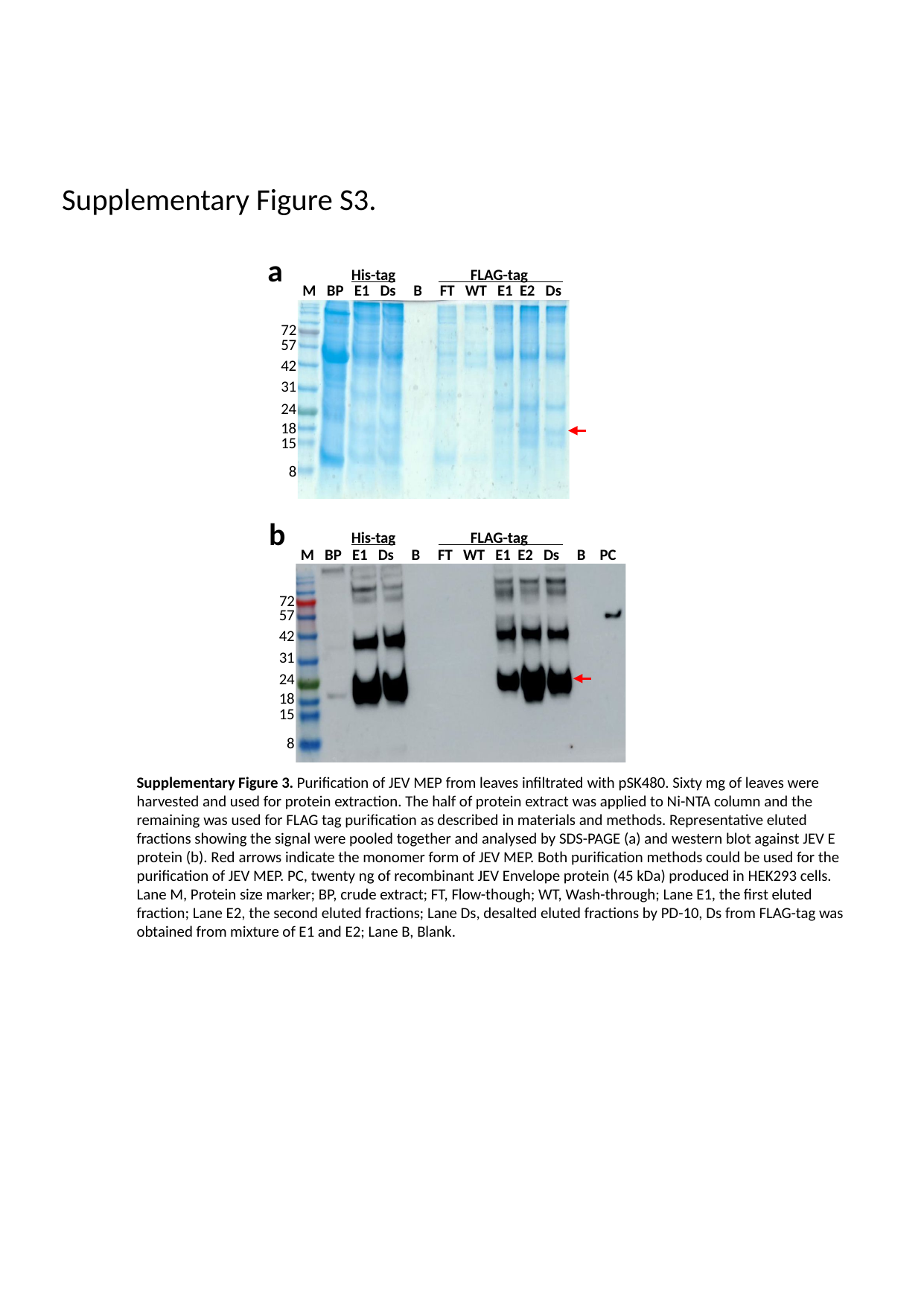

Supplementary Figure S3.
a
His-tag
 FLAG-tag .
M BP E1 Ds B FT WT E1 E2 Ds
72
57
42
31
24
18
15
8
b
His-tag
 FLAG-tag .
M BP E1 Ds B FT WT E1 E2 Ds B PC
72
57
42
31
24
18
15
8
Supplementary Figure 3. Purification of JEV MEP from leaves infiltrated with pSK480. Sixty mg of leaves were harvested and used for protein extraction. The half of protein extract was applied to Ni-NTA column and the remaining was used for FLAG tag purification as described in materials and methods. Representative eluted fractions showing the signal were pooled together and analysed by SDS-PAGE (a) and western blot against JEV E protein (b). Red arrows indicate the monomer form of JEV MEP. Both purification methods could be used for the purification of JEV MEP. PC, twenty ng of recombinant JEV Envelope protein (45 kDa) produced in HEK293 cells. Lane M, Protein size marker; BP, crude extract; FT, Flow-though; WT, Wash-through; Lane E1, the first eluted fraction; Lane E2, the second eluted fractions; Lane Ds, desalted eluted fractions by PD-10, Ds from FLAG-tag was obtained from mixture of E1 and E2; Lane B, Blank.

## Slide 4
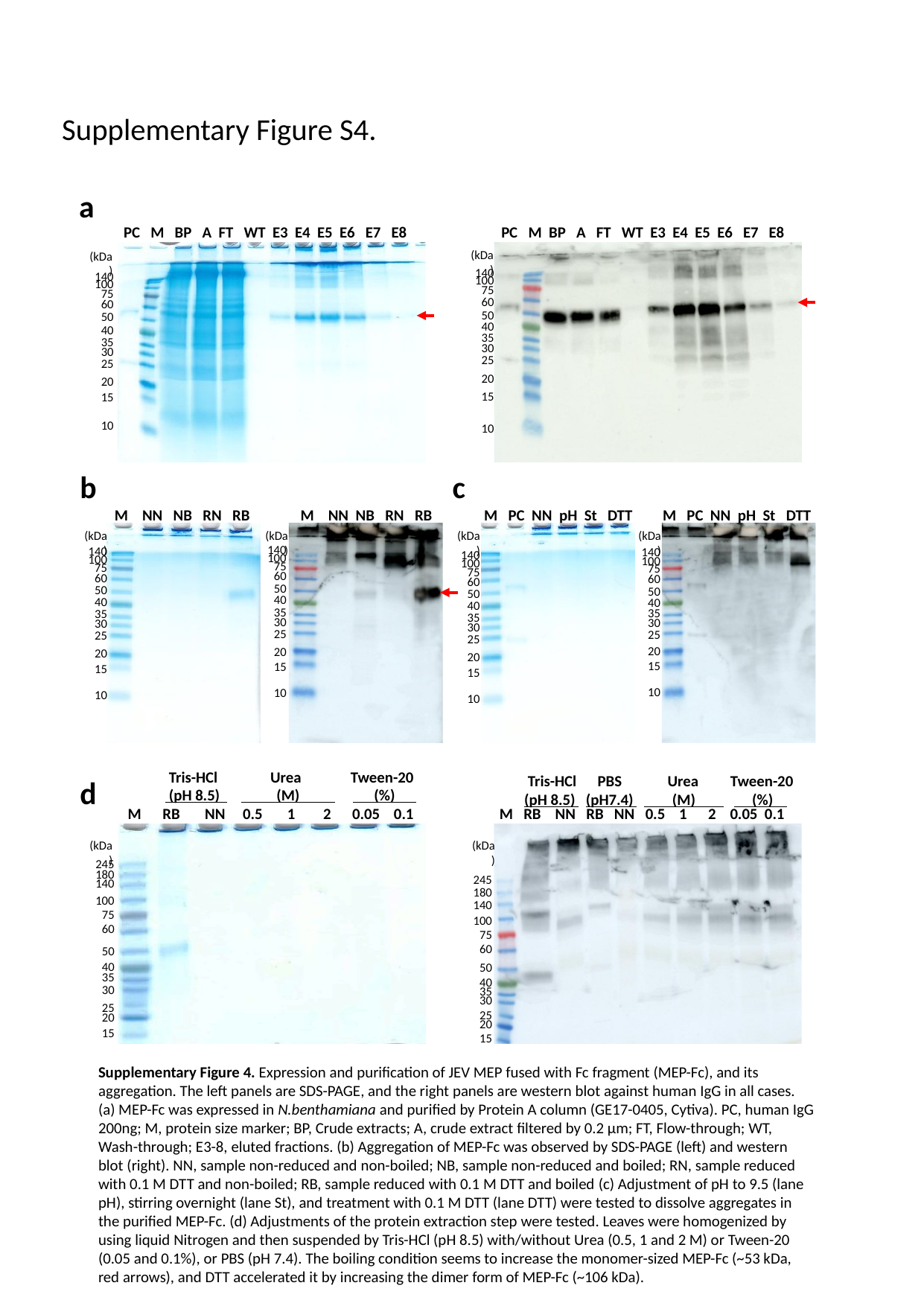

Supplementary Figure S4.
a
PC M BP A FT WT E3 E4 E5 E6 E7 E8
PC M BP A FT WT E3 E4 E5 E6 E7 E8
(kDa)
(kDa)
140
140
100
100
75
75
60
60
50
50
40
40
35
35
30
30
25
25
20
20
15
15
10
10
c
b
M PC NN pH St DTT
M NN NB RN RB
M PC NN pH St DTT
M NN NB RN RB
(kDa)
(kDa)
(kDa)
(kDa)
140
140
140
140
100
100
100
100
75
75
75
75
60
60
60
60
50
50
50
50
40
40
40
40
35
35
35
35
30
30
30
30
25
25
25
25
20
20
20
20
15
15
15
15
10
10
10
10
 Tris-HCl Urea Tween-20
 (pH 8.5) (M) (%) .
 Tris-HCl PBS Urea Tween-20
(pH 8.5) (pH7.4) (M) (%) .
d
M RB NN 0.5 1 2 0.05 0.1
M RB NN RB NN 0.5 1 2 0.05 0.1
5 sec
(kDa)
(kDa)
245
180
245
140
180
100
140
75
100
60
75
60
50
40
50
35
40
30
35
30
25
25
20
20
15
15
Supplementary Figure 4. Expression and purification of JEV MEP fused with Fc fragment (MEP-Fc), and its aggregation. The left panels are SDS-PAGE, and the right panels are western blot against human IgG in all cases. (a) MEP-Fc was expressed in N.benthamiana and purified by Protein A column (GE17-0405, Cytiva). PC, human IgG 200ng; M, protein size marker; BP, Crude extracts; A, crude extract filtered by 0.2 µm; FT, Flow-through; WT, Wash-through; E3-8, eluted fractions. (b) Aggregation of MEP-Fc was observed by SDS-PAGE (left) and western blot (right). NN, sample non-reduced and non-boiled; NB, sample non-reduced and boiled; RN, sample reduced with 0.1 M DTT and non-boiled; RB, sample reduced with 0.1 M DTT and boiled (c) Adjustment of pH to 9.5 (lane pH), stirring overnight (lane St), and treatment with 0.1 M DTT (lane DTT) were tested to dissolve aggregates in the purified MEP-Fc. (d) Adjustments of the protein extraction step were tested. Leaves were homogenized by using liquid Nitrogen and then suspended by Tris-HCl (pH 8.5) with/without Urea (0.5, 1 and 2 M) or Tween-20 (0.05 and 0.1%), or PBS (pH 7.4). The boiling condition seems to increase the monomer-sized MEP-Fc (~53 kDa, red arrows), and DTT accelerated it by increasing the dimer form of MEP-Fc (~106 kDa).

## Slide 5
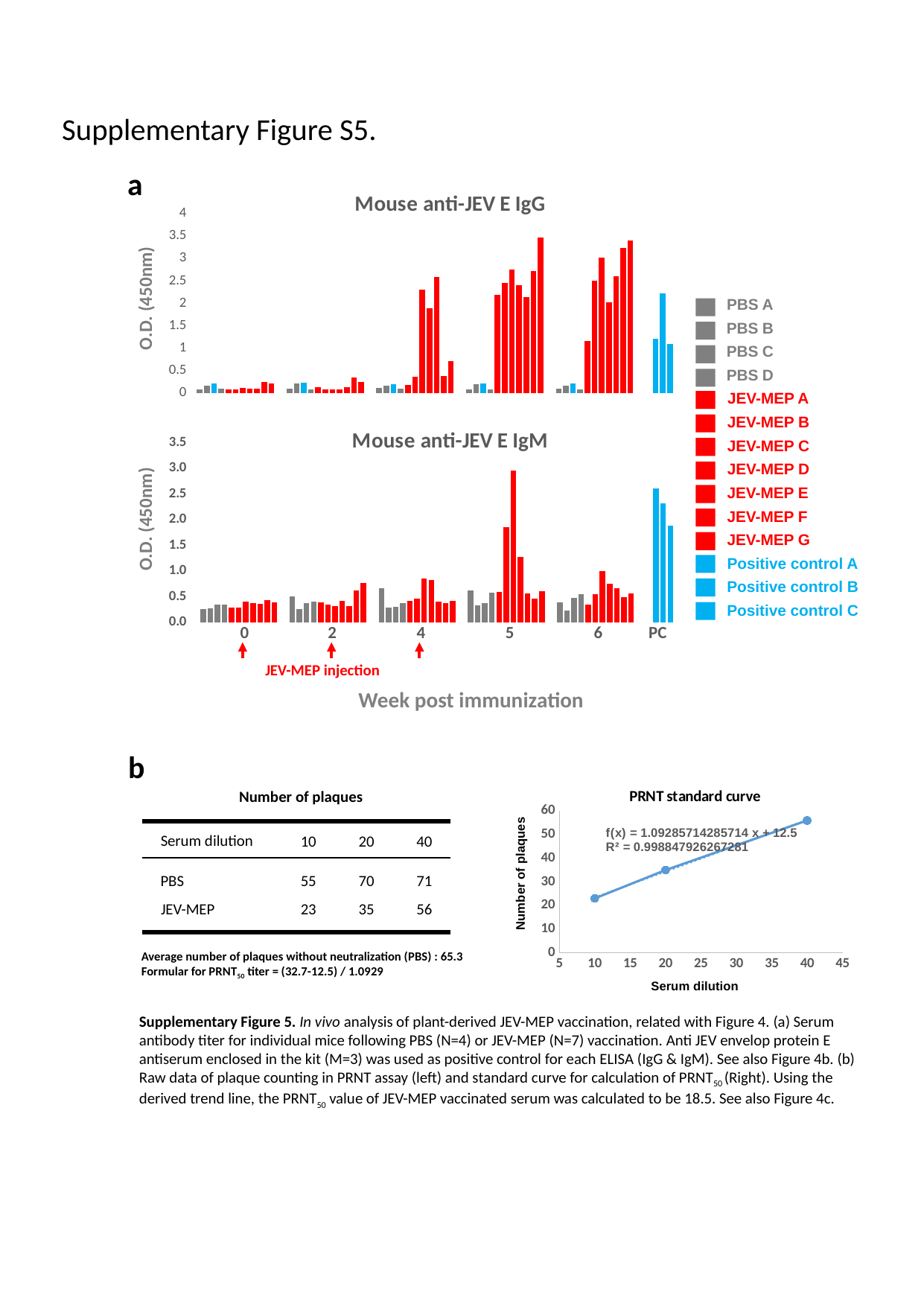

Supplementary Figure S5.
### Chart: Mouse anti-JEV E IgG
| Category | PBS | PBS | PBS | PBS | 20ug | 20ug | 20ug | 20ug | 20ug | 20ug | 20ug |
|---|---|---|---|---|---|---|---|---|---|---|---|
| 0 | 0.0923 | 0.1756 | 0.2098 | 0.096 | 0.0878 | 0.0837 | 0.1222 | 0.1083 | 0.1003 | 0.2541 | 0.2153 |
| 2 | 0.0976 | 0.2121 | 0.235 | 0.0906 | 0.1315 | 0.093 | 0.0912 | 0.0938 | 0.1398 | 0.3478 | 0.2577 |
| 4 | 0.1155 | 0.1709 | 0.2006 | 0.1043 | 0.1925 | 0.373 | 2.3109 | 1.8886 | 2.5818 | 0.3779 | 0.7078 |
| 5 | 0.0854 | 0.2063 | 0.2213 | 0.0889 | 2.1845 | 2.4468 | 2.7501 | 2.4004 | 2.1478 | 2.7232 | 3.4745 |
| 6 | 0.1016 | 0.1695 | 0.2125 | 0.0929 | 1.1565 | 2.4996 | 3.0234 | 2.0196 | 2.5993 | 3.232 | 3.3978 |
| P.C | None | 1.2055 | 2.2271 | 1.0946 | None | None | None | None | None | None | None |a
O.D. (450nm)
PBS A
PBS B
PBS C
PBS D
JEV-MEP A
### Chart: Mouse anti-JEV E IgM
| Category | PBS | PBS | PBS | PBS | 20ug | 20ug | 20ug | 20ug | 20ug | 20ug | 20ug |
|---|---|---|---|---|---|---|---|---|---|---|---|
| 0 | 0.2578 | 0.2765 | 0.3409 | 0.3428 | 0.2883 | 0.2852 | 0.4123 | 0.3798 | 0.3584 | 0.4319 | 0.3844 |
| 2 | 0.5037 | 0.2664 | 0.3744 | 0.4123 | 0.394 | 0.3464 | 0.3158 | 0.4173 | 0.3185 | 0.6175 | 0.7662 |
| 4 | 0.6679 | 0.2864 | 0.3 | 0.3781 | 0.4215 | 0.4662 | 0.855 | 0.8221 | 0.407 | 0.3801 | 0.4244 |
| 5 | 0.6299 | 0.3282 | 0.3835 | 0.5808 | 0.5924 | 1.8539 | 2.9628 | 1.2754 | 0.5702 | 0.4639 | 0.6145 |
| 6 | 0.3944 | 0.2295 | 0.4732 | 0.5557 | 0.3516 | 0.5538 | 0.9999 | 0.7574 | 0.6643 | 0.4943 | 0.5614 |
| P.C | None | 2.6179 | 2.3242 | 1.8822 | None | None | None | None | None | None | None |JEV-MEP B
JEV-MEP C
JEV-MEP D
JEV-MEP E
JEV-MEP F
O.D. (450nm)
JEV-MEP G
Positive control A
Positive control B
Positive control C
0
2
4
5
6
PC
JEV-MEP injection
Week post immunization
b
### Chart: PRNT standard curve
| Category | |
|---|---|Number of plaques
Serum dilution
10 20 40
Number of plaques
PBS
55 70 71
JEV-MEP
23 35 56
Average number of plaques without neutralization (PBS) : 65.3
Formular for PRNT50 titer = (32.7-12.5) / 1.0929
Serum dilution
Supplementary Figure 5. In vivo analysis of plant-derived JEV-MEP vaccination, related with Figure 4. (a) Serum antibody titer for individual mice following PBS (N=4) or JEV-MEP (N=7) vaccination. Anti JEV envelop protein E antiserum enclosed in the kit (M=3) was used as positive control for each ELISA (IgG & IgM). See also Figure 4b. (b) Raw data of plaque counting in PRNT assay (left) and standard curve for calculation of PRNT50 (Right). Using the derived trend line, the PRNT50 value of JEV-MEP vaccinated serum was calculated to be 18.5. See also Figure 4c.
