# Supplementary material for: Production of Plant-Derived Japanese Encephalitis Virus Multi-Epitope Peptide in Nicotiana benthamiana and Immunological Response in Mice
Source: Int J Mol Sci. 2023 Jul 19;24(14):11643. doi: 10.3390/ijms241411643 (PMC10380836; doi:10.3390/ijms241411643)

Figure 2a

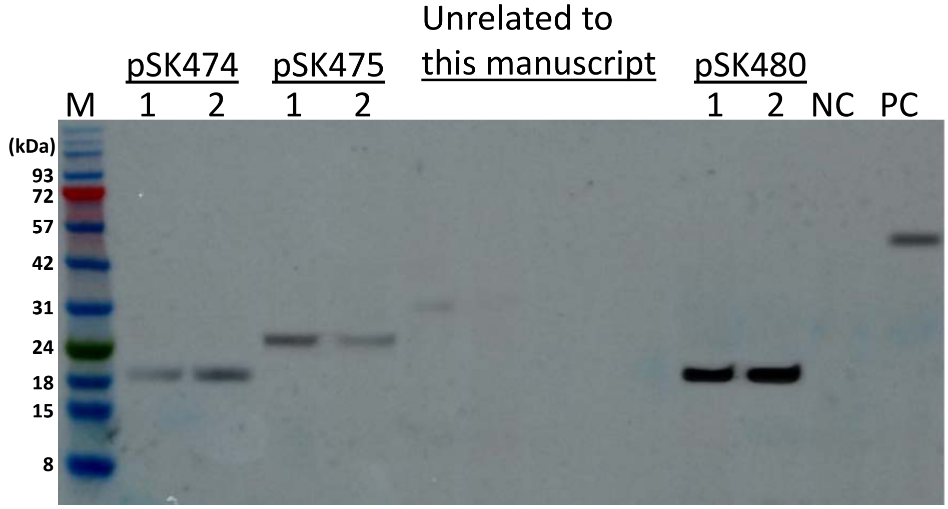

Figure 2b

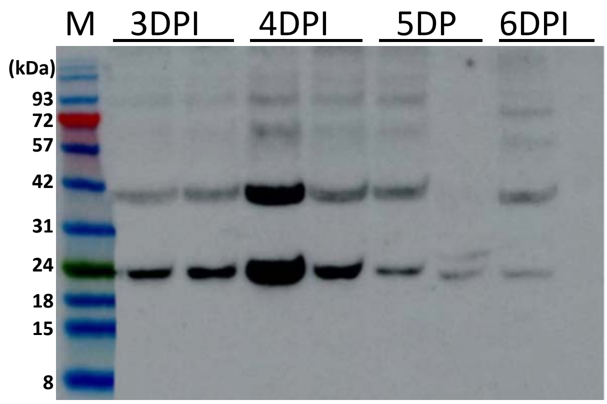

Figure 2c

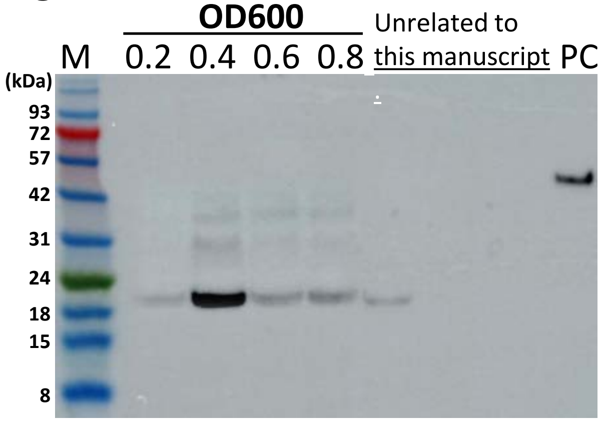

Figure 2d

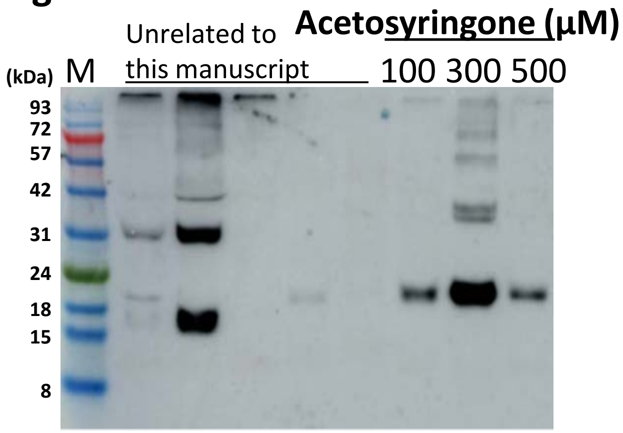

Figure 2e

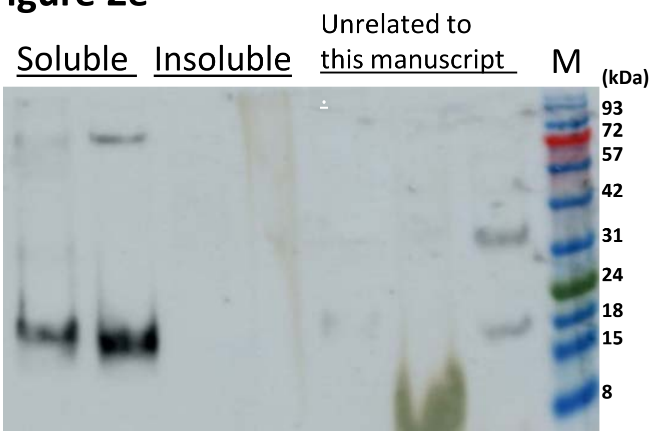

Figure 3 (left)

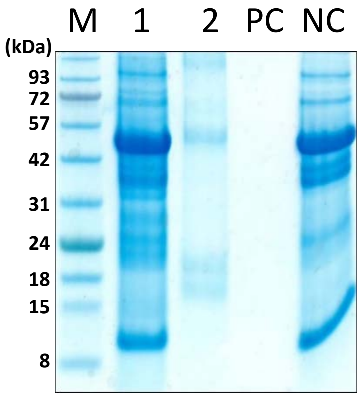

Figure 3 (right)

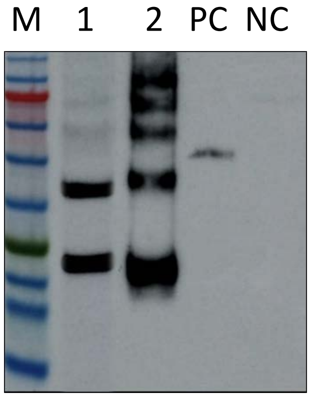

Supplementary figure S2

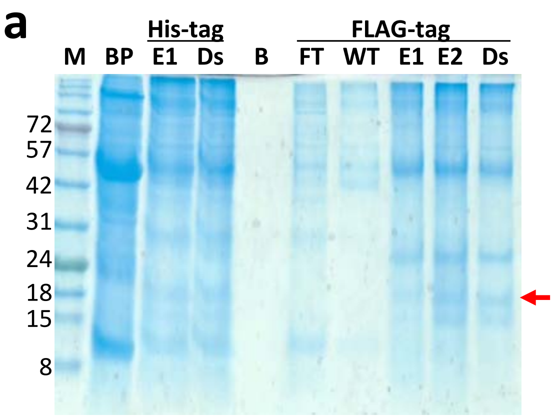

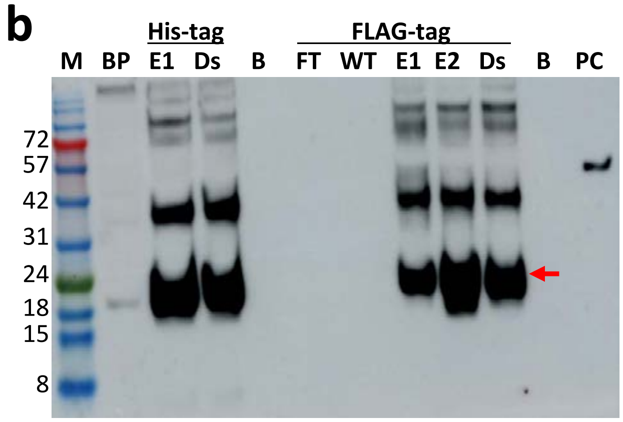

Supplementary figure S3.

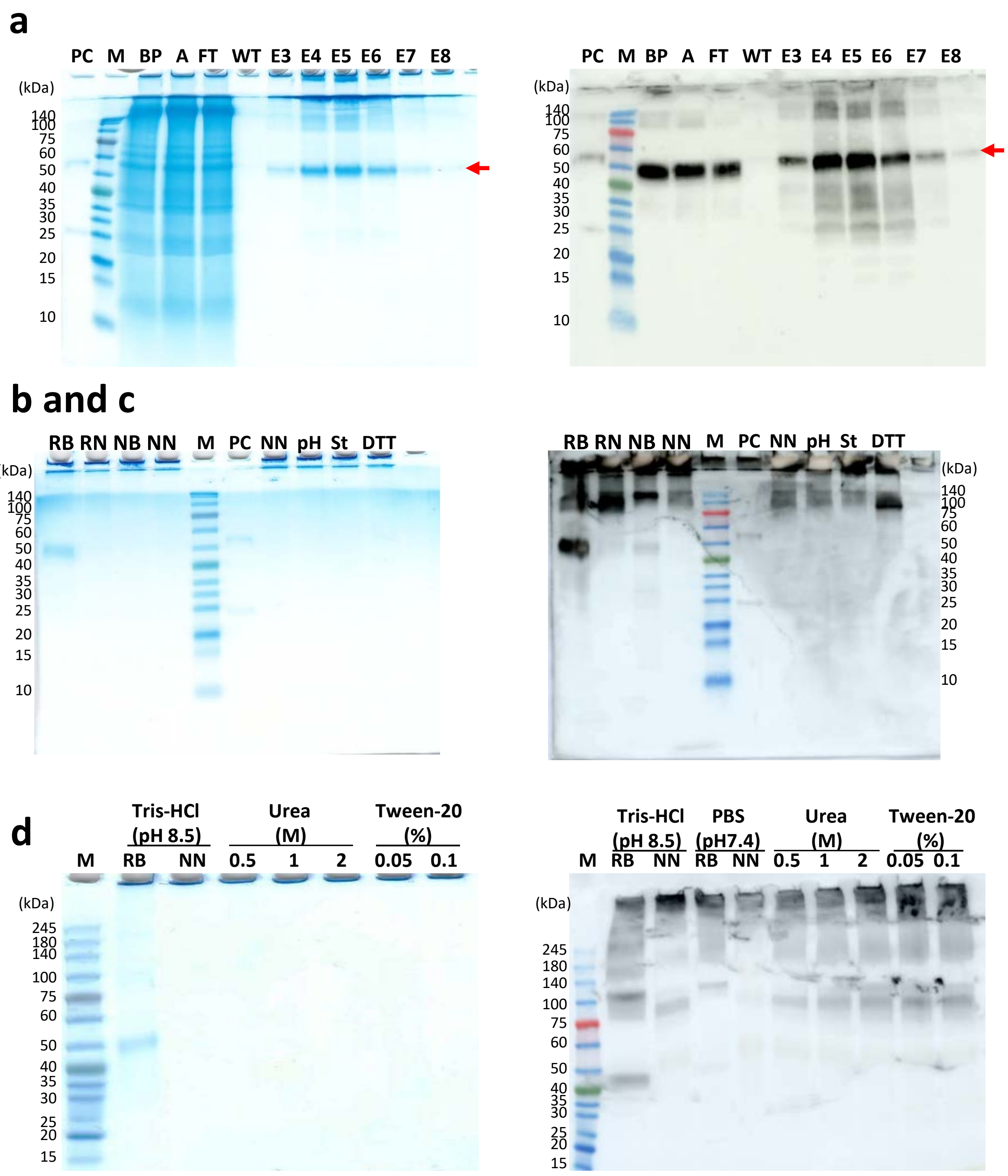

Supplement: Supplementary file 1 [file ijms-24-11643-s001.zip › Original figures.pdf]
